# A Prediction Model with Lifestyle in Addition to Previously Known Risk Factors Improves Its Predictive Ability for Cardiovascular Death

**DOI:** 10.1038/s41598-019-49003-5

**Published:** 2019-09-10

**Authors:** Masatoshi Nishimoto, Miho Tagawa, Masaru Matsui, Masahiro Eriguchi, Ken-ichi Samejima, Kunitoshi Iseki, Chiho Iseki, Koichi Asahi, Kunihiro Yamagata, Tsuneo Konta, Shouichi Fujimoto, Ichiei Narita, Masato Kasahara, Yugo Shibagaki, Toshiki Moriyama, Masahide Kondo, Tsuyoshi Watanabe, Kazuhiko Tsuruya

**Affiliations:** 1 Department of Nephrology, Nara Medical University, Nara, Japan; 2 Steering Committee of Research on Design of the Comprehensive Health Care System for Chronic Kidney Disease (CKD) Based on the Individual Risk Assessment by Specific Health Check, Fukushima, Japan

**Keywords:** Lifestyle modification, Risk factors

## Abstract

This longitudinal cohort study aimed to create a novel prediction model for cardiovascular death with lifestyle factors. Subjects aged 40–74 years in the Japanese nationwide Specific Health Checkup Database in 2008 were included. Subjects were randomly assigned to the derivation and validation cohorts by a 2:1 ratio. Points for the prediction model were determined using regression coefficients that were derived from the Cox proportional hazards model in the derivation cohort. Models 1 and 2 were developed using known risk factors and known factors with lifestyle factors, respectively. The models were validated by comparing Kaplan-Meier curves between the derivation and validation cohorts, and by calibration plots in the validation cohort. Among 295,297 subjects, data for 120,823 were available. There were 310 cardiovascular deaths during a mean follow-up of 3.6 years. Model 1 included known risk factors. In model 2, weight gain, exercise habit, gait speed, and drinking alcohol were additionally included as protective factors. Kaplan-Meier curves matched better between the derivation and validation cohorts in model 2, and model 2 was better calibrated. In conclusion, our prediction model with lifestyle factors improved the predictive ability for cardiovascular death.

## Introduction

The Framingham Heart Study identified several risk factors for coronary heart disease (CHD) and the Framingham Risk Score (FRS) was developed^1^. The FRS is widely known as a standard tool for the 10-year probability of the incidence of CHD. Since this development, several important prediction models for CHD^2^ or cardiovascular (CV) events^3–8^ have been developed. These models have been used to identify high-risk patients, for developing preventive measures for those with a high risk, or for determining a high-risk population for clinical trials for intervention or prevention of CV events.

To the best of our knowledge, only a few studies have directly reported a prediction model for CV death^9–11^. A risk chart was developed from the SCORE project^9^ that predicts the 10-year probability of all CV death in men and women. Variables included in this risk chart are age, systolic blood pressure (SBP), smoking habit, and serum total cholesterol (TC) levels or the TC/high-density lipoprotein cholesterol (HDL-C) ratio. NIPPON DATA80^10^ also investigated predictors of the 10-year probability of CV death in Japanese people. These predictors included serum glucose levels in addition to variables that were included in the SCORE project. However, these studies did not consider variables that were recently reported to be associated with CV events, such as the estimated glomerular filtration rate (eGFR)^2,12^ or proteinuria^13–16^. Furthermore, no studies have developed a model with various lifestyle factors. Sedentary lifestyle is closely associated with metabolic syndrome^17^, and subsequently with CV events^18^. Some of the lifestyles, such as gait speed, may reflect frailty. Gait speed is associated not only with all-cause mortality^19,20^, but also with CV death^21^.

This study aimed to develop a novel prediction model for CV death that is composed of previously known factors and lifestyle factors, and to compare this model with a model only including previously known risk factors.

## Methods

### Study design and subjects

This was a longitudinal study based on a database from the Specific Health Checkup program. Details of this cohort have been published previously^22^. In 2008, the Japanese government started a new annual health check program (Specific Health Checkup) to support early diagnosis and intervention in metabolic syndrome. This study was part of an ongoing project called “Research on design of the comprehensive health care system for chronic kidney disease (CKD) based on individual risk assessment by Specific Health Checkup for all Japanese citizens aged 40–74 years old.” Databases that were included in this study were from Fukushima, Ibaraki, Osaka, Fukuoka, Miyazaki, and Okinawa Prefecture, which indicates regional diversity. Subjects described age, sex, smoking status, past history of stroke, and CHD in questionnaires. For physical data, trained staff measured height, body weight (BW), and blood pressure (BP) using a standard sphygmomanometer or an automated device on the right arm after resting for 5 min in the sitting position. For laboratory data, blood samples were collected after fasting overnight. Centrifuged samples were analyzed by an automatic clinical chemistry analyzer within 24 h. All blood samples were analyzed at local, rather than central, laboratories. Low-density lipoprotein cholesterol (LDL-C) levels were directly measured and not calculated by the Friedewald method^23^. The results of a urine dipstick test for proteinuria were interpreted by the medical staff and recorded as −, +/−, 1+, 2+, or 3+. Analyses were conducted by the methods for laboratory tests as recommended by the Japan Society of Clinical Chemistry. The database also included answers to lifestyle questionnaires. Details of the questionnaires are shown in Supplemental Table S1. Data were sent to a data center called the NPO Japan Clinical Research Support Unit to be verified. Outliers with extremely high values, 10 times higher than normal range, were deleted through winsorization and accounted for 0.01% to 0.1% of the total.

The inclusion criterion was subjects aged 40–74 years old in the database. The exclusion criterion was subjects with missing data for variables for analyses.

Ethical approval was obtained from respective institutional review board and the steering committee approved the protocol. The data were completely de-identified before being provided to the investigators.

### Exposures of interests and outcomes

Variables that were included in the prediction models derived from the Framingham Heart Study^1^, the Suita Study^2^, the SCORE project^9^, NIPPON DATA 80^10^, and the BioBank Japan project^11^ were included as previously known risk factors. These variables were age, sex, body mass index (BMI), history of stroke, history of CHD, current smoking, diabetes mellitus (DM), BP, eGFR, HDL-C, TC, and LDL-C. Proteinuria was also included in previously known risk factors because associations between proteinuria and CV diseases have been reported^13–16^. Answers to questionnaires on lifestyles were included in model 2. The outcome variable was CV death. Those who died because of CV diseases were identified using the national database of death certificates from 2008–2012, and the causes of death were classified according to International Classification of Diseases 10.

### Definitions

BMI was calculated as BW divided by the square of height in meters. Current smoking was defined as smoking more than 100 cigarettes in total or any for longer than 6 months and continued until the last month. Stroke was defined as self-reported hemorrhagic and ischemic stroke, and CHD as self-reported angina pectoris or myocardial infarction. DM was determined by American Diabetes Association criteria^24^ as follows: fasting plasma glucose levels ≥7.0 mmol/L (126 mg/dL), hemoglobin A1c National Glycohemoglobin Standardization Program ≥6.5%, or prescription of antidiabetic agents. The eGFR was calculated using a formula that was developed for the Japanese population: eGFR (males) = 194 × serum creatinine (Scr)^−1.094^ × age^−0.287^ and eGFR (females) = eGFR (males) × 0.739^25^.

The causes of death were classified into the following categories according to ICD-10 codes; death due to stroke: I60.0, I60.6, I60.9, I61.0, I61.3, I61.4, I61.6, I61.9, I62.0, I62.9, I63.2, I63.4, I63.8, I63.9, I64, I67.9, I69.1, I69.3, death due to CHD: I20.9, I21.0, I21.9, I24.9, I25.1, I25.8, I25.9, death due to aortic dissection or rupture of an aneurysm: I71.0, I71.1, I71.2, I71.3, I71.8, acute cardiac death: I46.0, I46.1, I46.9, fatal arrhythmia: I47.2, I48, I49.0, I49.4, I49.8, I49.9, heart failure: I50.0, I50.9, and others: I10, I11.9, I12.0, I26.9, I27.0, I31.9, I33.0, I35.0, I38, I42.0, I42.2, I51.4, I51.5, I51.9, I74.3, I80.2, I81, I84.1.

### Statistical analyses

Subjects were randomly assigned to the derivation and validation cohorts by a 2:1 ratio. Data were shown as mean (SD) or number (%). Models 1 and 2 were developed using previously known risk factors and previously known risk factors plus lifestyle factors, respectively. The Cox proportional hazard model was used to develop prediction models for CV death. Continuous variables were divided into categories as follows. Age was divided into 40–49 years, 50–59 years, 60–69 years, and 70–74 years. BMI was divided into <18.5 kg/m^2^, ≥18.5 kg/m^2^ and <25.0 kg/m^2^, and ≥25.0 kg/m^2^. BP was divided into SBP <130 mm Hg and diastolic blood pressure (DBP) <85 mm Hg, SBP of 130 to 139 mm Hg or DBP of 85 to 89 mm Hg, SBP of 140 to 159 mm Hg or DBP of 90 to 99 mm Hg, and SBP ≥160 or DBP ≥100 mm Hg. The eGFR was divided into ≥60 mL/min/1.73 m^2^ and <60 mL/min/1.73 m^2^. Proteinuria was divided into − or +/− and + or more. HDL-C levels were divided into ≤0.89 mmol/L (34 mg/dL), 0.90 to 1.28 mmol/L (35 to 49 mg/dL), 1.29 to 1.54 mmol/L (50 to 59 mg/dL), and ≥1.55 mmol/L (60 mg/dL). LDL-C levels were divided into ≤1.80 mmol/L (69 mg/dL), 1.81 to 2.58 mmol/L (70 to 99 mg/dL), 2.59 to 3.61 mmol/L (100 to 139 mg/dL), and ≥3.62 mmol/L (140 mg/dL). TC levels were divided into ≤4.13 mmol/L (159 mg/dL), 4.14 to 6.20 mmol/L (160 to 239 mg/dL), 6.21 to 7.23 mmol/L (240 to 279 mg/dL), and ≥7.24 mmol/L (280 mg/dL). The frequency of drinking was divided into 2 categories of no or rarely and sometimes or every day. BP was classified according to the FRS^1^, and when SBP and DBP fell into different categories, a higher category was selected. HDL-C, LDL-C, and TC levels were basically classified according to the FRS^1^, and Japanese Circulation Society guideline^26^. Variables were selected using stepwise backward elimination by the likelihood ratio test in the derivation cohort. Among variables that were selected by backward elimination, only those that were significantly associated with CV death were included in the prediction model. For a level of significance of backward selection, p value < 0.05 was used. Points in the models were generated by dividing each regression coefficient by the smallest absolute value of the regression coefficient in the prediction model and rounding up to the nearest integer.

The created models were validated by following two methods^27^. The derivation and validation cohorts were divided into 3 categories (low, middle, and high risk for CV death) according to points in the prediction models and Kaplan-Meier curves were compared between the derivation and validation cohorts. The cut-off points between 3 risk groups were selected per each model where there were step-ups of the incidence rates of CV death.

In the validation cohort, the 3-year predicted probability of CV death was calculated using baseline survival function and regression coefficients in the derivation cohort. Calibration plots were created by ranking subjects into deciles of predicted probability and plotting the predicted and observed incidence in each group. Two models were compared by calibration slopes and R^2^ rather than Hosmer-Lemeshow chi-square test, as the incidence rate of CV death was extremely low, which violated the chi-square test assumption^28^.

Statistical analyses were performed using the STATA software program version 15 (STATA Corp., College Station, TX) and SPSS version 23.0 (IBM Corp., Armonk, NY).

### Ethics approval and consent to participate

All procedures performed in studies involving human participants were in accordance with the ethical standards of the institutional and/or national research committee at which the studies were conducted (Fukushima Medical University; IRB Approval Number #1485, #2771) and with the 1964 Helsinki declaration and its later amendments or comparable ethical standards. This study was conducted according also to the Ethical Guidelines for Medical and Health Research Involving Human Subjects enacted by the Ministry of Health, Labour and Welfare of Japan [http://www.mhlw.go.jp/file/06-Seisakujouhou-10600000-Daijinkanboukouseikagakuka/0000069410.pdf]. In the context of the guideline, the investigators shall not necessarily be required to obtain informed consent, but we made public information concerning this study on the web [http://www.fmu.ac.jp/univ/sangaku/data/koukai_2/2771.pdf] and ensured the opportunities for the research subjects to refuse utilizing their personal information.

## Results

**Figure 1:**
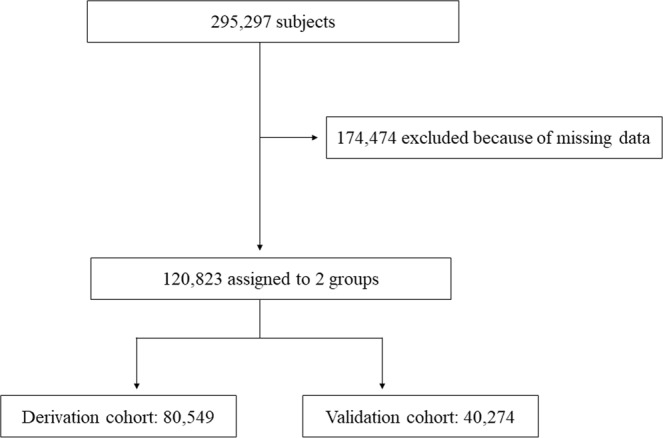
Flow chart of the subjects.

Among 295,297 subjects, data for 120,823 were available for analyses. There were no significant differences in demographics between included and excluded subjects (data not shown). Included subjects were divided into the derivation and validation cohorts by a 2:1 ratio (n = 80,549 and 40,274, respectively) (Fig. 1). The demographics of the subjects are shown in Table 1.

**Table 1:** Characteristics of Subjects in the Derivation and Validation Cohorts

	Derivation cohort (n = 80,549)	Validation cohort (n = 40,274)	Standardized difference, %
Age (years)	63.9 (8.2)	63.9 (8.2)	−1.1
Male sex	32,987 (41.0)	16,465 (40.9)	−0.1
BMI, kg/m^2^	23.4 (3.3)	23.4 (3.3)	0.5
History of stroke	2992 (3.7)	1509 (3.7)	0.2
History of CHD	4946 (6.1)	2404 (6.0)	−0.7
Current smoking	10,447 (13.0)	5040 (12.5)	−1.4
DM	7554 (9.4)	3778 (9.4)	<0.01
SBP, mm Hg	129.3 (17.7)	129.2 (17.7)	0.7
DBP, mm Hg	76.3 (10.7)	76.2 (10.8)	0.7
Proteinuria	4571 (5.7)	2325 (5.8)	0.4
eGFR, mL/min/1.73 m^2^	74.5 (15.8)	74.5 (16.1)	−0.1
HDL-C, mmol/L mg/dL	1.58 (0.41) 61.1 (15.7)	1.59 (0.41) 61.2 (15.8)	−0.3
TC, mmol/L mg/dL	5.45 (0.90) 210.5 (34.6)	5.45 (0.90) 210.6 (34.8)	−0.2
LDL-C, mmol/L mg/dL	3.26 (0.79) 125.7 (30.4)	3.26 (0.80) 125.8 (30.7)	−0.2
Weight gain ≥10 kg since 20 years old	28,753 (35.7)	14,418 (35.8)	0.2
Exercise habit	35,514 (44.1)	17,721 (44.0)	−0.2
Walking habit	41,829 (51.9)	20,905 (51.9)	−0.05
Gait speed (fast)	40,375 (50.1)	20,091 (49.9)	−0.5
Change in weight ≥3 kg/year	18,890 (23.5)	9332 (23.2)	−0.7
Eating speed (fast)	22,799 (28.3)	11,339 (28.2)	−0.3
Eating before bed	13,759 (17.1)	6743 (16.7)	−0.9
Snack	10,664 (13.2)	5332 (13.2)	<0.01
Skipping breakfast	8310 (10.3)	4072 (10.1)	−0.7
Drinking alcohol (Sometimes or every day)	36,305 (45.1)	18,051 (44.8)	−0.5
Enough sleep	60,739 (75.4)	30,561 (75.9)	1.1

Data are shown as number (%) or mean (SD). BMI: body mass index, CHD: coronary heart disease, DM: diabetes mellitus, SBP: systolic blood pressure, DBP: diastolic blood pressure, eGFR: estimated glomerular filtration rate, HDL-C: high-density lipoprotein cholesterol, TC: total cholesterol, LDL-C: low-density lipoprotein cholesterol, CV: cardiovascular.

During a mean follow-up period of 3.60 years, the incidence of CV death was 0.70 events/1000 person-years and 0.73 events/1000 person-years in the derivation and validation cohorts, respectively. The components of CV death are shown in Table 2.

**Table 2:** Causes of Death in the Derivation and Validation Cohorts.

Components	Derivation cohort (n = 80,549)	Validation cohort (n = 40,274)
Death due to stroke	74	33
Death due to CHD	49	36
Other CV death		
Aortic dissection or rupture of an aneurysm Acute cardiac death Fatal arrhythmia Heart failure Others	28 13 14 7 19	9 9 5 4 10
Total CV death	204	106
Non-CV death	766	424
All-cause death	970	530

Data are shown as number in each cohort. CHD: coronary heart disease, CV: cardiovascular.

The crude hazard ratios for CV death associated with each variable were shown in Supplemental Table S2. Multivariable cox regression analyses were performed to identify predictors of CV death. Variables selected by backward elimination are shown in Tables 3 and 4. Among these variables, those that were significantly associated with CV death were included in the prediction models. Variables included in model 1 were as follows: age of 60–69 years, 3 points; age 70–74 years, 4 points; male sex, 1 point; BMI <18.5 kg/m^2^, 2 points; history of stroke, 1 point; history of CHD, 2 points; current smoking, 2 points; DM, 1 point; BP (SBP ≥160 or DBP ≥100 mm Hg), 3 points; proteinuria, 1 point; and eGFR <60 mL/min/1.73 m^2^, 1 point. Variables included in model 2 were as follows: age of 60–69 years, 4 points; age 70–74 years, 5 points; male sex, 2 points; history of CHD, 2 points; current smoking, 2 points; DM, 2 points; BP (SBP ≥160 or DBP ≥100 mm Hg), 4 points; proteinuria, 2 points; eGFR <60 mL/min/1.73 m^2^, 1 point; weight gain ≥10 kg since 20 years old, −1 point; exercise habit, −1 point; fast gait speed, −1 point; and drinking alcohol (sometimes or every day), −1 point.

**Table 3:** Model 1: Cox Regression with Previously Known Risk Factors and Points in the Prediction Model (Derivation Cohort).

	Category	β	Points	*P*	HR	95% CI
Age, years	40–49 (ref)	—				
	50–59	0.31	—	0.56	1.36	0.49–3.79
	60–69	1.13	3	0.02	3.10	1.25–7.72
	70–74	1.62	4	0.001	5.03	2.00–12.63
Sex	Male	0.58	1	<0.001	1.78	1.31–2.42
BMI, kg/m^2^	≥18.5 and <25.0 (ref)	—				
	<18.5	0.69	2	0.02	2.00	1.12–3.57
	≥25	0.15	—	0.33	1.16	0.86–1.56
History of stroke	Yes	0.51	1	0.04	1.67	1.04–2.67
History of CHD	Yes	0.81	2	<0.001	2.26	1.55–3.29
Current smoking	Yes	0.77	2	<0.001	2.15	1.53–3.03
DM	Yes	0.53	1	0.003	1.70	1.20–2.41
Blood pressure, mm Hg	SBP <130 and DBP <85 (ref)	—				
	SBP of 130 to 139 or DBP of 85 to 89	0.30	—	0.13	1.35	0.92–1.98
	SBP of 140 to 159 or DBP of 90 to 99	0.32	—	0.10	1.37	0.94–2.00
	SBP ≥160 or DBP ≥100	1.38	3	<0.001	3.96	2.65–5.91
Proteinuria	(+), (++), or (+++)	0.57	1	0.005	1.77	1.19–2.63
eGFR, mL/min/1.73 m^2^	<60	0.41	1	0.01	1.51	1.09–2.07

Variables were selected by stepwise backward elimination and variables that were significantly associated with cardiovascular death were included in the prediction model.

Points were generated by dividing each regression coefficient by the smallest absolute value of the regression coefficient in the prediction model, and rounding up to the nearest integer.

ref: reference, BMI: body mass index, CHD: coronary heart disease, DM: diabetes mellitus, SBP: systolic blood pressure, DBP: diastolic blood pressure, eGFR: estimated glomerular filtration rate, β: regression coefficient.

**Table 4:** Model 2: Cox Regression with Previously Known Risk Factors and Lifestyle Factors, and Points in the Prediction Model (Derivation Cohort).

	Category	β	Points	*P*	HR	95% CI
Age, years	40–49 (ref)	—				
	50–59	0.32	—	0.55	1.37	0.49–3.82
	60–69	1.19	4	0.01	3.27	1.31–8.14
	70–74	1.66	5	<0.001	5.24	2.08–13.20
Sex	Male	0.78	2	<0.001	2.19	1.56–3.07
BMI, kg/m^2^	≥18.5 and <25.0 (ref)	—				
	<18.5	0.55	—	0.06	1.73	0.97–3.10
	≥25	0.33	—	0.06	1.39	0.99–1.94
History of stroke	Yes	0.43	—	0.07	1.54	0.96–2.48
History of CHD	Yes	0.79	2	<0.001	2.20	1.51–3.21
Current smoking	Yes	0.72	2	<0.001	2.05	1.45–2.89
DM	Yes	0.54	2	0.003	1.71	1.21–2.42
Blood pressure, mm Hg	SBP <130 and DBP <85 (ref)	—				
	SBP of 130 to 139 or DBP of 85 to 89	0.33	—	0.10	1.39	0.95–2.04
	SBP of 140 to 159 or DBP of 90 to 99	0.36	—	0.07	1.43	0.98–2.08
	SBP ≥160 or DBP ≥100	1.41	4	<0.001	4.08	2.73–6.10
Proteinuria	(+), (++), or (+++)	0.53	2	0.009	1.70	1.14–2.53
eGFR, mL/min/1.73 m^2^	<60	0.42	1	0.01	1.53	1.11–2.10
Weight gain ≥10 kg since 20 years old	Yes	−0.46	−1	0.008	0.63	0.45–0.89
Exercise habit	Yes	−0.45	−1	0.003	0.64	0.48–0.86
Gait speed	Fast	−0.38	−1	0.01	0.69	0.51–0.92
Drinking alcohol	Sometimes or every day	−0.32	−1	0.04	0.72	0.53–0.99

Variables were selected by stepwise backward elimination and variables that were significantly associated with cardiovascular death were included in the prediction model.

Points were generated by dividing each regression coefficient by the smallest absolute value of the regression coefficient in the prediction model, and rounding up to the nearest integer.

ref: reference, BMI: body mass index, CHD: coronary heart disease, DM: diabetes mellitus, SBP: systolic blood pressure, DBP: diastolic blood pressure, eGFR: estimated glomerular filtration rate, β: regression coefficient.

**Figure 2:**
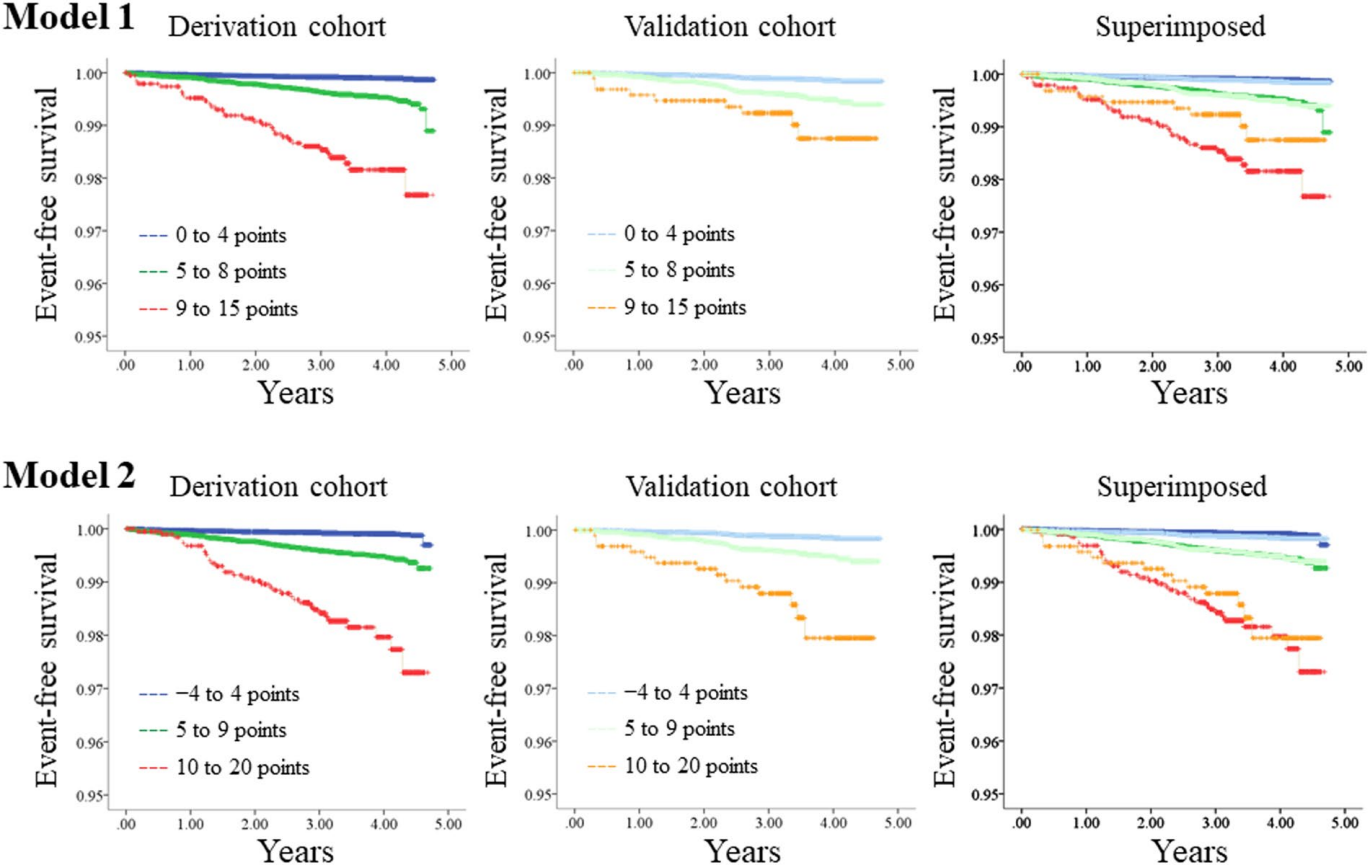
Kaplan-Meier curves for cardiovascular death. Cohorts were divided into 3 risk groups according to points in prediction models. Kaplan-Meier curves for the 3 groups were compared between the derivation and validation cohorts.

**Figure 3:**
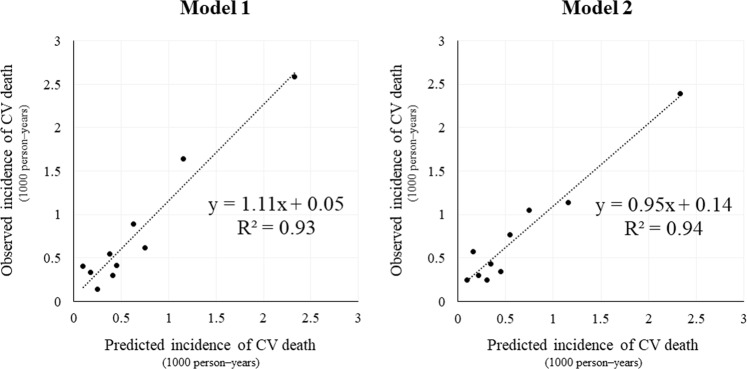
Calibration plots for the incidence of CV death within 3 years in the validation cohort. Subjects were divided into deciles by each predicted probability, and predicted and observed incidence of CV death within 3 years were plotted.

The distribution of points in each prediction model is shown in Supplemental Fig. S1. Actual incidence rates of CV death for subjects with each point are shown in Table 5. According to incidence rates of CV death, subjects were divided into 3 risk categories as follows for model 1: low risk, 0 to 4 points; middle risk, 5 to 8 points; and high risk, 9 to 15 points. Subjects were divided into 3 risk categories as follows for model 2: low risk, −4 to 4 points; middle risk, 5 to 9 points; and high risk, 10 to 20 points. Kaplan-Meier curves were compared between the derivation and validation cohorts (Fig. 2). There was deviation in Kaplan-Meier curves between the derivation and validation cohorts among the high risk group in model 1, but the 3 curves matched better in model 2. The calibration plots for CV death within 3 years for models 1 and 2 are shown in Fig. 3. The calibration slope was y = 1.11x + 0.05 (R^2^ = 0.93) in model 1 and y = 0.95x + 0.14 (R^2^ = 0.94) in model 2, which suggested that model 2 was calibrated better. As a sensitivity analysis, we also created calibration plots restricted to those without a history of CHD or stroke. The calibration slope was y = 1.21x − 0.02 (R^2^ = 0.91) in model 1 and y = 0.88x + 0.12 (R^2^ = 0.88) in model 2, suggesting that our model also worked well for those without history of CV events.

**Table 5:**
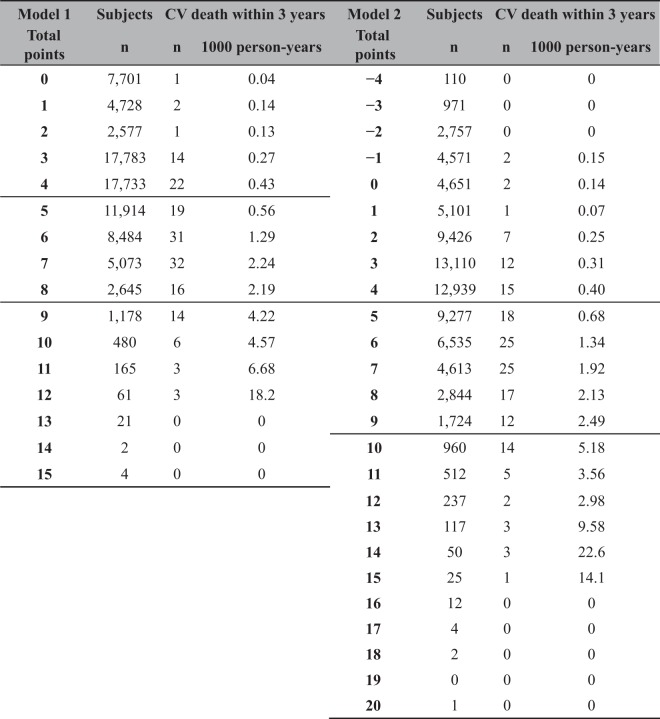
The Number of Subjects and Incidence of CV Death within 3 Years According to the Total Points for Both Prediction Models (Derivation Cohort).

The cohort was divided into 3 risk categories according to the incidence of CV death as follows for model 1: low risk, 0 to 4 points; middle risk, 5 to 8 points; and high risk, 9 to 15 points. The cohort was divided into 3 risk categories according to the incidence of CV death as follows for model 2: low risk, −4 to 4 points; middle risk, 5 to 9 points; and high risk 10 to 20 points.

CV: cardiovascular.

## Discussion

In this study, 2 prediction models for CV death were developed and compared. Model 1 was composed of previously known risk factors for CV events. In model 2, an additional 4 lifestyle components (weight gain ≥10 kg since 20 years old, exercise habit, fast gait speed, and drinking alcohol) were included. Model 2 showed improved predictive ability for CV death compared with model 1.

Our prediction models were different from previous models in several points. Our model 1 considered variables (age, sex, BMI, history of stroke, history of CHD, current smoking, DM, BP, eGFR, HDL-C, TC, LDL-C) that were derived from the Framingham Heart Study^1^, the Suita Study^2^, the SCORE project^9^, NIPPON DATA 80^10^, and the BioBank Japan project^11^, as well as proteinuria. These variables remained in model 1, except for cholesterol levels. The results did not change when TC or LDL-C was separately included in the model or different cut-off points were used. There are 2 possible explanations for why cholesterol was not associated with CV death in this study. First, aggressive cholesterol lowering has been recommended in recent guidelines in patients with a high risk for CV events. Therefore, patients with a high risk for CV events tend to have low TC and LDL-C levels. The FRS^1^ and Suita Score^2^, where LDL-C and TC were predictors of CHD, were based on a cohort that was recruited in the 1970s or 1990s. At that time, the target for lipid control was not so strict as that currently used. In our database, subgroup analysis in subjects who were not taking lipid-lowering agents showed that the incidence of death due to CHD tended to be higher in those with LDL-C levels ≥2.59 mmol/L (100 mg/dL) (hazard ratio 1.99 [95% confidence interval: 0.95–4.18]). This result suggests that LDL-C levels are a risk factor for CHD in patients who are not taking lipid-lowering agents. However, aggressive lipid lowering in high risk subgroups masked the association between LDL-C and CV death. The second possibility is that the association between LDL-C and CV events could be different from the association between LDL-C and CV death. The BioBank Japan database, which was registered in the 2000s, showed that lower TC levels, especially <4.66 mmol/L (180 mg/dL), were associated with CV death in subjects with chronic phase CV disease^11^. In our study, lower BMI, especially <18.5 kg/m^2^, was also associated with CV death. These results suggest that those with high LDL-C levels have a higher risk for CV events. However, those who develop CV events, despite having low LDL-C levels and BMI, and they are likely to have malnutrition, are more prone to death.

Our model 1 included the eGFR and proteinuria. The Suita study reported that the prediction model for CHD, which included an eGFR <60 mL/min/1.73 m^2^, was superior to the FRS in Japanese patients^2^. Although proteinuria was not included in previous prediction models for CV events, multiple studies have reported an association between proteinuria and CV events^13–16^. The data from the Second National Health and Nutrition Examination Survey (NHANES II) and the NHANES II Mortality Study indicated that urinary protein levels of 30 mg/dL to 299 mg/dL and ≥300 mg/dL are more associated with CV death compared with urinary protein levels <30 mg/dL (*P* = 0.02)^13^. A community-based cohort study in Canada also showed that, compared with normal protein levels, mild (urine dipstick trace or 1+) or heavy (urine dipstick ≥2+) proteinuria is associated with a higher incidence of myocardial infarction within every eGFR stratum^15^. According to the HOPE study, albuminuria was an independent risk factor for CV events, and the risk proportionally increased with the urine albumin to creatinine ratio, regardless of the presence of DM^29^. Furthermore, a meta-analysis demonstrated that the eGFR and proteinuria were multiplicatively associated with the risk of cardiovascular mortality^16^. In our study, eGFR and proteinuria were independent predictors of CV death and included in the prediction models.

Our model 2 was unique in that it included lifestyle factors. Weight gain ≥10 kg since 20 years old, exercise habit, fast gait speed, and drinking alcohol were included in this model. Unexpectedly, we found that weight gain ≥10 kg since 20 years old was associated with a lower incidence of CV death. Excessive weight gain is associated with the incidence of type 2 DM^30^. However, a modest weight gain is consistently associated with the lowest all-cause mortality rate, and long-term weight loss, even of a mild or moderate degree, is associated with a higher mortality rate^31^. In our study, weight gain ≥10 kg since 20 years old was not associated with CV death in univariate analysis. However, when BMI was included in the covariates, it was significantly associated with a lower incidence of CV death. In model 1, low BMI was associated with a higher incidence of CV death, whereas in model 2, BMI was excluded from the model. The association between weight gain and a lower incidence of CV death is probably a reflection of the association between weight loss and a higher incidence of CV death. With regard to gait speed, data from NHANES 1999–2004 showed an association between slow gait speed, especially <0.8 m/s, and all-cause mortality^20^. According to a systematic review, 6 of 7 studies also showed that slow gait speed was associated with CV death^21^. With regard to exercise habit, the term “exercising to sweat lightly” indicated approximately 4 metabolic equivalents. Each 1 metabolic equivalent increase in exercise capacity conferred a 12% improvement in survival^32^. Exercise improves control of hypertension^33^ or DM^34^. Exercise might be a link between fast gait speed or exercise habit and a lower incidence of CV death. Additionally, those with unrecognized CHD might unintentionally protect themselves from experiencing angina by limiting physical activity and a slow gait speed might be a reflection of underlying unrecognized CV disease. Drinking alcohol (answering yes to “drinking sometimes or every day”) was associated with a lower incidence of CV death in our study. In our study, 64.3% of all drinkers consumed <20 g of alcohol, and 88.3% drank <40 g/day. Previous studies have consistently reported a J-shaped or U-shaped curve for the association between alcohol use and all-cause mortality or CV death. A small amount of alcohol intake (10–20 g/day for women and 20–40 g/day for men) was associated with lower all-cause mortality^35^. In another study, overall CV death was lower in subjects who reported consuming at least one drink daily compared with non-drinkers, despite the highest all-cause mortality among heavy drinkers in a large trial that included 490,000 subjects^36^. Suggested mechanisms for the benefit of moderate alcohol consumption are as follows: an increase in HDL-C levels, improvement in insulin sensitivity, favorable effects mediated by alterations in protein kinase, anti-inflammatory effects, an increase in adiponectin levels, an increase in fibrinolysis, a decrease in platelet aggregation and coagulation, and improved endothelial function^37^.

This study has several strengths compared with previous studies. First, model 2 is unique in that it included 4 lifestyle factors. Addition of lifestyle factors improved the predictive ability for CV death. Questionnaires on lifestyle could be administered without any cost and could be useful in identifying those with a high risk for CV death. Second, a large number of subjects were included. The results of our study have generalizability because the subjects were from an unselected general population. Third, the outcome was CV death, which was a hard endpoint. CV death must have been accurate because it was ascertained by linking the database to the national database of death certificates.

Our study also has a few limitations. Because this was an observational study, associations that were observed in the study do not imply causal relationships. An example of this limitation is that fast gait speed was associated with a lower incidence of CV death. However, whether walking fast prevents CV death or a slow gait speed is an indicator of unrecognized CV diseases is unknown. Additionally, lifestyle factors were based on the answers to questionnaires and not based on objective assessment.

## Conclusions

A novel prediction model for CV death was developed using lifestyle factors in addition to previously known risk factors for CV death. This model performs better than the model with previously known risk factors alone. Clinical implications of our prediction model require further investigation. External validation in different populations is also required to confirm the validity of our new model.

## Supplementary information

Supplementary materials

## Data Availability

The dataset used in this study is not publicly available due to the restriction by the agreement among the research group members.
