# Supplementary material for: A Prediction Model with Lifestyle in Addition to Previously Known Risk Factors Improves Its Predictive Ability for Cardiovascular Death
Source: Sci Rep. 2019 Sep 10;9:12953. doi: 10.1038/s41598-019-49003-5 (PMC6736867; doi:10.1038/s41598-019-49003-5)
Supplement: Supplementary file 1 — Supplementary materials [file 41598_2019_49003_MOESM1_ESM.docx]

**A Prediction Model with Lifestyle in Addition to Previously Known Risk Factors Improves Its Predictive Ability for Cardiovascular Death**

Masatoshi Nishimoto, MD^1^; Miho Tagawa, MD, PhD^1^; Masaru Matsui, MD, PhD^1^; Masahiro Eriguchi, MD, PhD^1^; Ken-ichi Samejima, MD, PhD^1^; Kunitoshi Iseki, MD, PhD^2^; Chiho Iseki, PhD^2^; Koichi Asahi, MD, PhD^2^; Kunihiro Yamagata, MD, PhD^2^; Tsuneo Konta, MD, PhD^2^; Shouichi Fujimoto, MD, PhD^2^; Ichiei Narita, MD, PhD^2^; Masato Kasahara, MD, PhD^2^; Yugo Shibagaki, MD, PhD^2^; Toshiki Moriyama, MD, PhD^2^; Masahide Kondo, MD, PhD^2^; Tsuyoshi Watanabe, MD, PhD^2^; Kazuhiko Tsuruya*, MD, PhD^1,2^

1. Department of Nephrology, Nara Medical University, Nara, Japan.

2. Steering Committee of Research on Design of the Comprehensive Health Care System for Chronic Kidney Disease (CKD) Based on the Individual Risk Assessment by Specific Health Check, Fukushima, Japan.

**Corresponding author:** Kazuhiko Tsuruya, MD, PhD

Department of Nephrology, Nara Medical University

840 Shijocho, Kashihara, Nara, 634-8521, Japan

E-mail: [tsuruya@naramed-u.ac.jp](mailto:tsuruya@naramed-u.ac.jp), Tel: +81-744-29-8859, Fax: +81-744-23-9913

**Table S1. Questionnaires on Lifestyle**

| **Weight gain ≥10 kg since 20 years old** | I have gained ≥10 kg since 20 years old. |
| --- | --- |
| **Exercise habit** | I have been exercising to sweat lightly at least twice a week, at least 30 min per session, for at least 1 year. |
| **Walking habit** | I have been walking or doing the same level of exercise every day for at least 1 hour. |
| **Gait speed** | I tend to walk faster than other people of the same age and sex. |
| **Change in weight** | I have gained or lost ≥3 kg in the past year. |
| **Eating speed** | I tend to eat faster than other people. |
| **Eating before bed** | I eat dinner within 2 hours of going to sleep 3 times or more a week. |
| **Snack** | I eat snacks after dinner 3 times or more a week. |
| **Skipping breakfast** | I skip breakfast 3 times or more a week. |
| **Drinking alcohol** | I sometimes drink alcohol or drink alcohol every day, and I am not a non-drinker. |
| **Enough sleep** | I get enough sleep to be rested every day. |

**Table S2. The crude hazard ratios for CV death associated with each variable in the derivation cohort**

|  | **Category** | **Hazard Ratio**  **(95% Confidence Interval)** |
| --- | --- | --- |
| Age, years | 40–49 (ref) | – |
|  | 50–59 | 1.36 (0.49–3.78) |
|  | 60–69 | 3.38 (1.38–8.30) |
|  | 70–74 | 6.00 (2.43–14.8) |
| Sex | Male | 2.61 (1.96–3.47) |
| BMI, kg/m^2^ | ≥18.5 and <25.0 (ref) | – |
|  | <18.5 | 1.51 (0.85–2.68) |
|  | ≥25 | 1.50 (1.09–1.95) |
| History of stroke | Yes | 2.92 (1.84–4.63) |
| History of CHD | Yes | 3.30 (2.29–4.75) |
| Current smoking | Yes | 2.20 (1.61–3.03) |
| DM | Yes | 2.72 (1.95–3.79) |
| Blood pressure, mm Hg | SBP <130  and  DBP <85 (ref) | – |
|  | SBP of 130 to 139  or  DBP of 85 to 89 | 1.67 (1.14–2.43) |
|  | SBP of 140 to 159  or  DBP of 90 to 99 | 1.81 (1.25–2.62) |
|  | SBP ≥160  or  DBP ≥100 | 5.47 (3.71–8.08) |
| Proteinuria | (+), (++), or (+++) | 3.27 (2.25–4.74) |
| eGFR, mL/min/1.73 m^2^ | <60 | 2.29 (1.69–3.11) |
| HDL-C, mmol/L  mg/dL | ≤0.89  ≤34 (ref) | – |
|  | 0.90–1.28  35–49 | 0.61 (0.28–1.34) |
|  | 1.29–1.54  50–59 | 0.46 (0.21–1.00) |
|  | ≥1.55  ≥60 | 0.36 (0.17–0.78) |
| TC, mmol/L  mg/dL | ≤4.13  ≤159 (ref) | – |
|  | 4.14–6.20  160–239 | 0.68 (0.41–1.12) |
|  | 6.21–7.23  240–279 | 0.47 (0.25–0.88) |
|  | ≥7.24  ≥280 | 0.79 (0.33–1.90) |
| LDL-C, mmol/L  mg/dL | ≤1.80  ≤69 (ref) | – |
|  | 1.81–2.58  70–99 | 0.42 (0.20–0.85) |
|  | 2.59–3.61  100–139 | 0.54 (0.28–1.02) |
|  | ≥3.62  ≥140 | 0.38 (0.19–0.74) |
| Weight gain ≥10 kg since 20 years old | Yes | 0.87 (0.65–1.16) |
| Exercise habit | Yes | 0.71 (0.53–0.95) |
| Walking habit | Yes | 0.71 (0.54–0.94) |
| Gait speed | Fast | 0.59 (0.45–0.79) |
| Change in weight ≥3 kg/year | Yes | 1.22 (0.90–1.66) |
| Eating speed | Fast | 0.83 (0.61–1.14) |
| Eating before bed | Yes | 0.95 (0.66–1.38) |
| Snack | Yes | 0.78 (0.50–1.21) |
| Skipping breakfast | Yes | 1.07 (0.69–1.65) |
| Drinking alcohol | Sometimes or every day | 1.07 (0.82–1.41) |


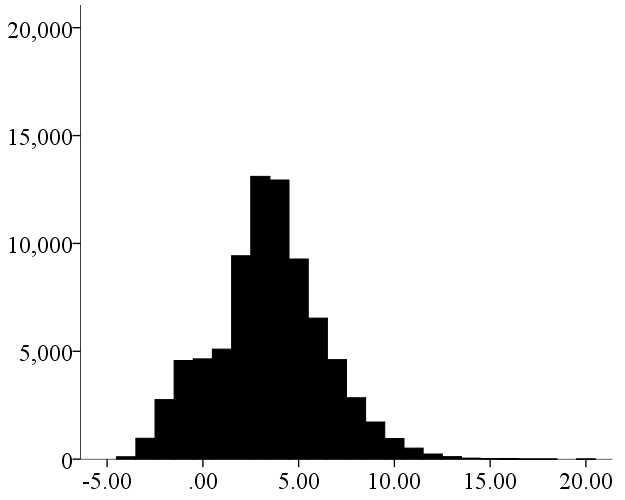

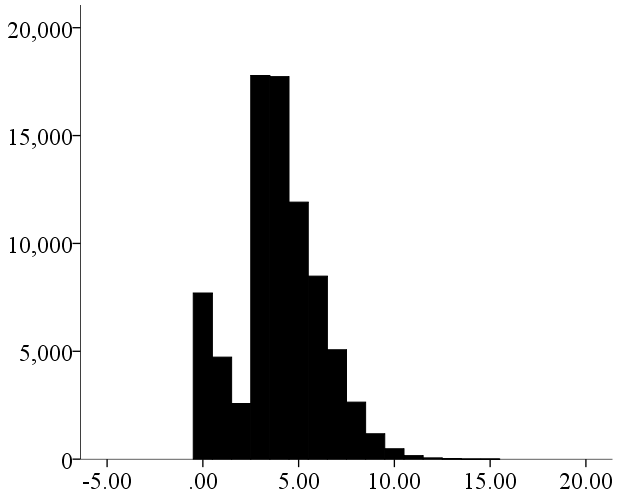


Model 2

Total points

Model 1

Total points

Number of subjects

Number of subjects

**Figure S1. Distribution of total points in the prediction models.**
